# Supplementary material for: Quantum Hall coherent perfect absorption in graphene
Source: Sci Rep. 2023 Dec 12;13:22076. doi: 10.1038/s41598-023-49191-1 (PMC10716163; doi:10.1038/s41598-023-49191-1)
Supplement: Supplementary file 1 — Supplementary Figures. [file 41598_2023_49191_MOESM1_ESM.pdf]

Quantum Hall coherent perfect absorption in graphene

Dariush Jahani,[1] Mohammadreza Alikhani,[1] and Yaser Abdi*[1]

[1] *Department of Physics. University of Tehran, Tehran, Iran*

Due to the extended primary results, the outputs are presented in this file.

I. TRANSMISSION AND REFLECTION

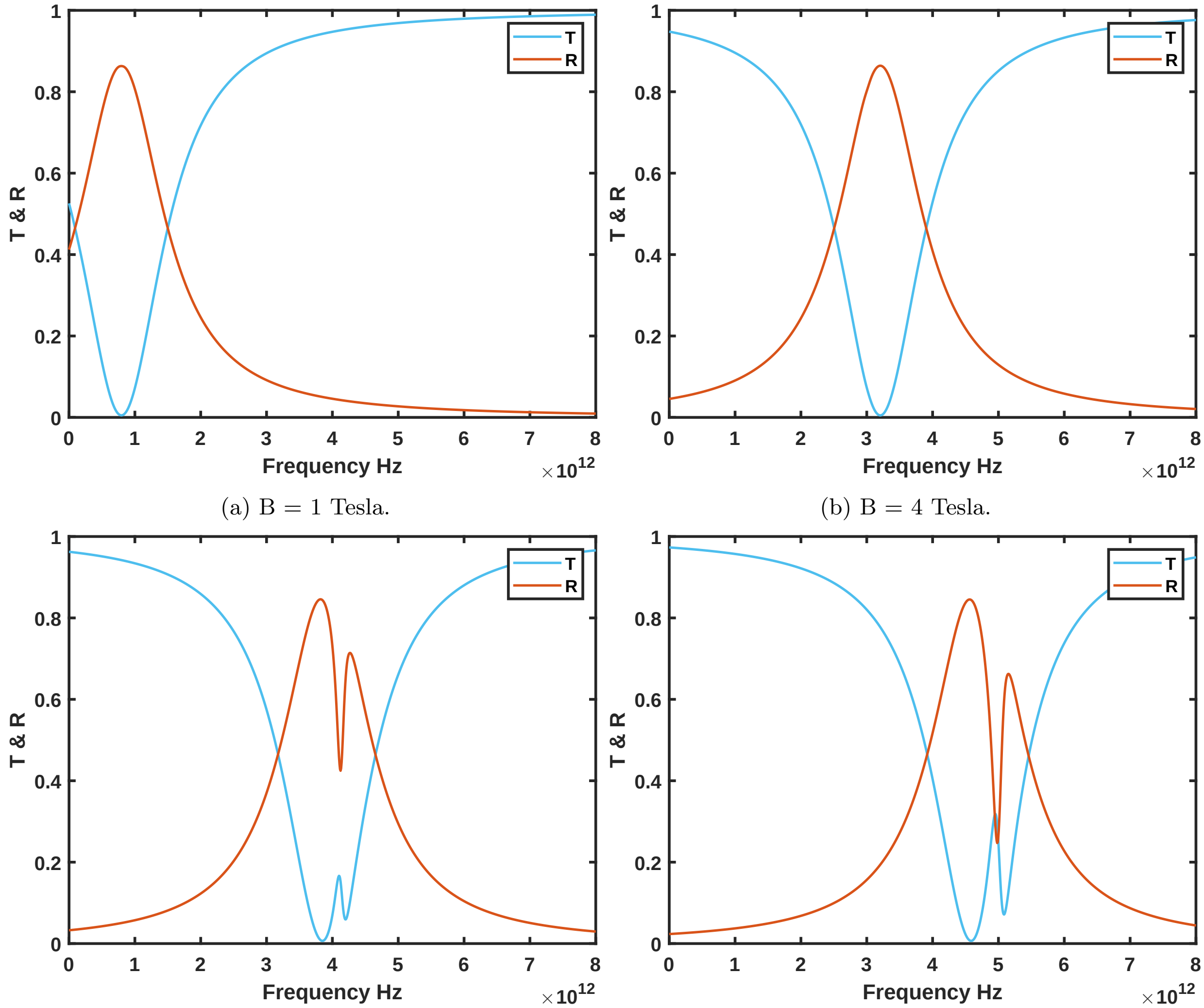

(a) B = 1 Tesla.

(b) B = 4 Tesla.

(c) B = 5 Tesla.

(d) B = 6 Tesla.

Supplementary figure S1: Transmission and reflection for left handed polarization, chemical potential: $0.2ev$, $T =$ 10K

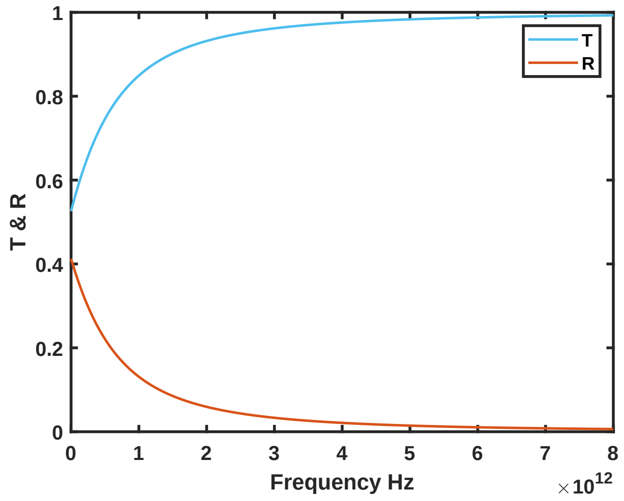

(a) B = 1 Tesla.

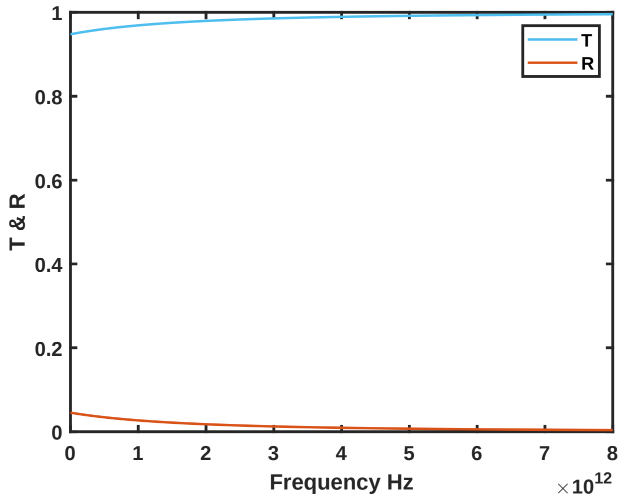

(b) B = 4 Tesla.

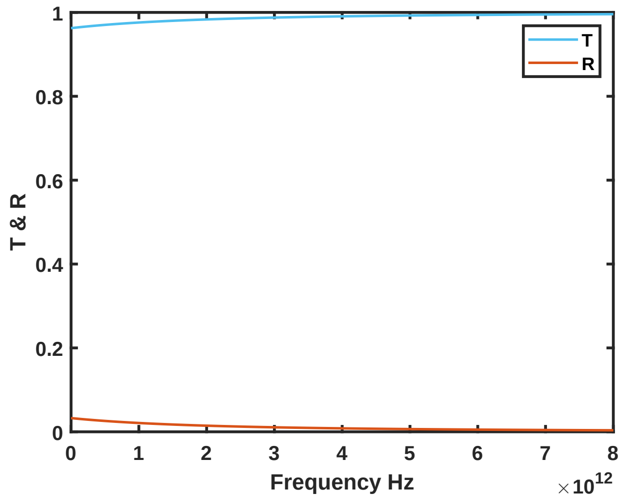

(c) B = 5 Tesla.

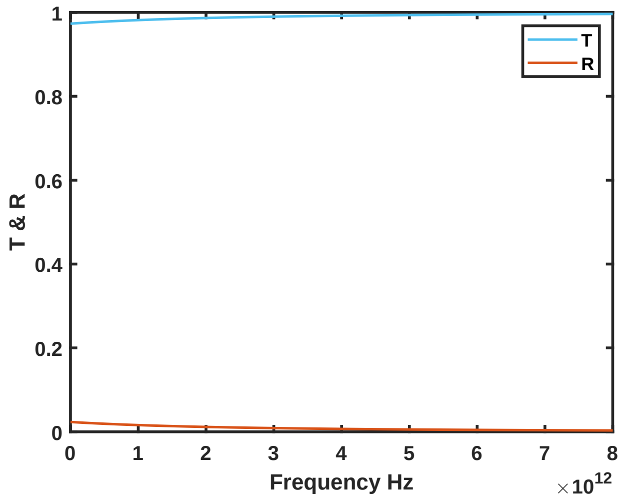

(d) B = 6 Tesla.

Supplementary figure S2: Transmission and reflection for right handed polarization, chemical potential: $0.2ev$, $T =$ 10K

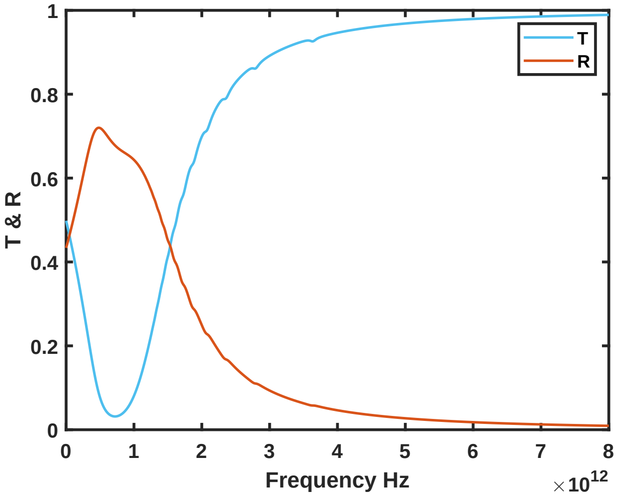

(a) B = 1 Tesla.

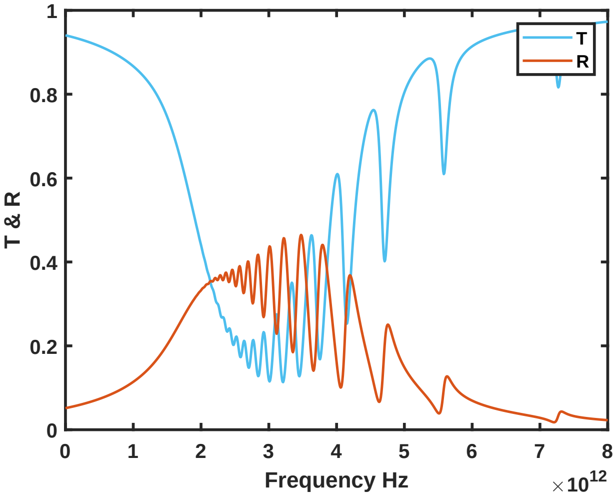

(b) B = 4 Tesla.

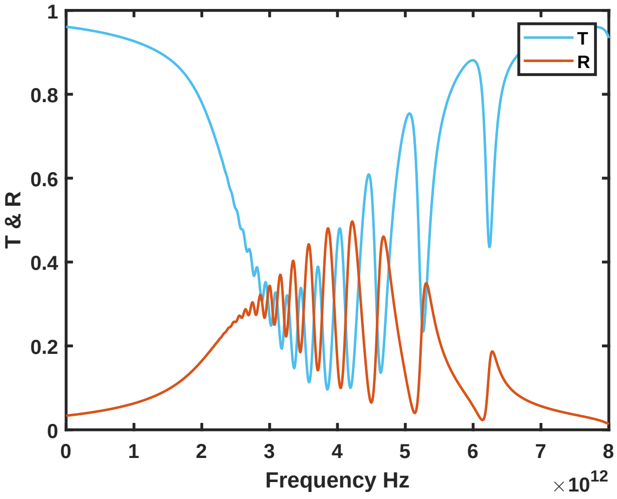

(c) B = 5 Tesla.

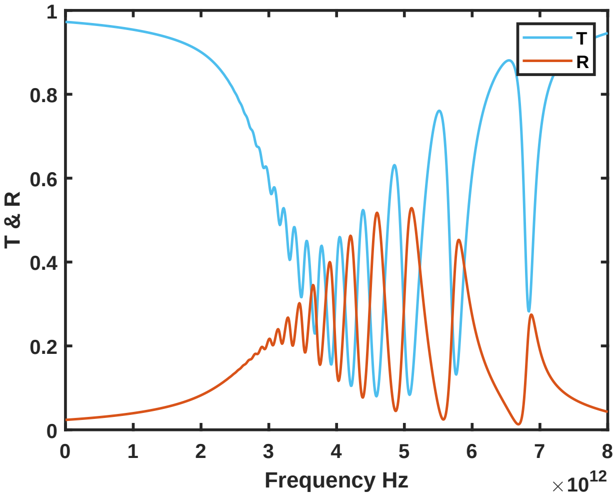

(d) B = 6 Tesla.

Supplementary figure S3: Transmission and reflection for left handed polarization, chemical potential: $0.2ev$, $T =$ 300K

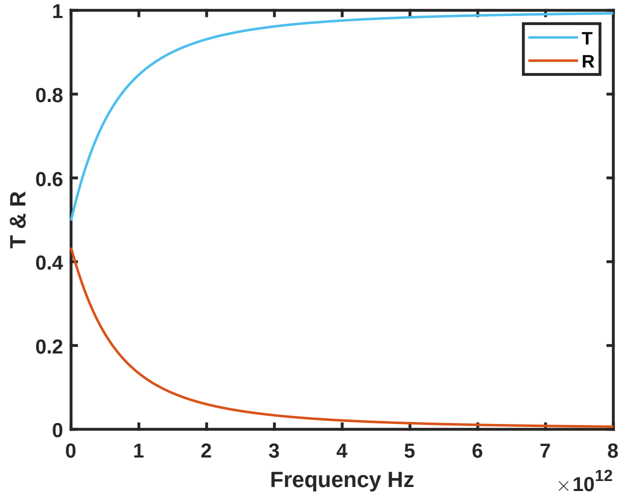

(a) B = 1 Tesla.

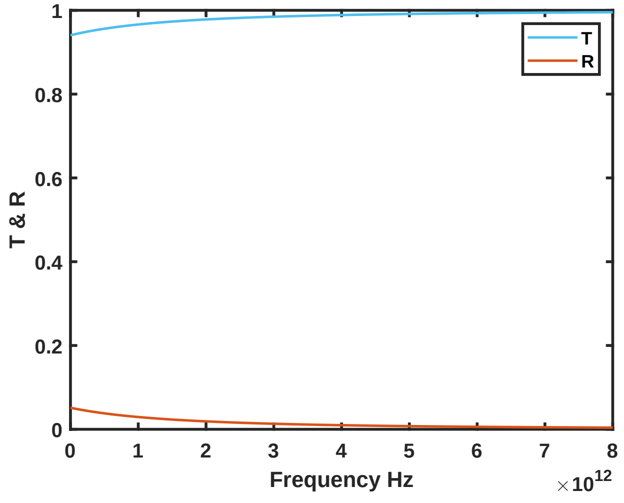

(b) B = 4 Tesla.

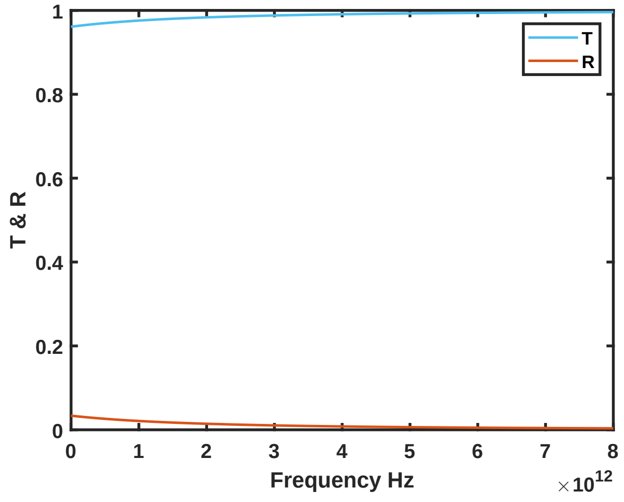

(c) B = 5 Tesla.

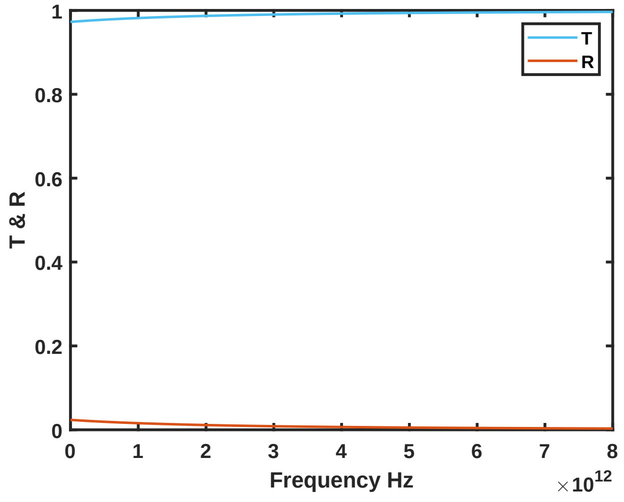

(d) B = 6 Tesla.

Supplementary figure S4: Transmission and reflection for right handed polarization, chemical potential: $0.2ev$, $T =$ 300K

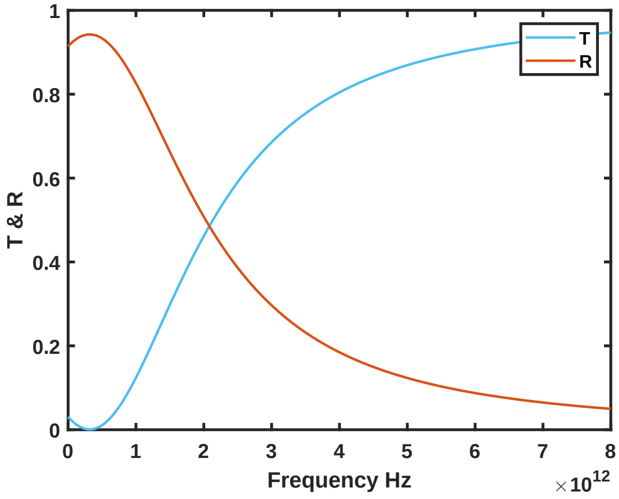

(a) B = 1 Tesla.

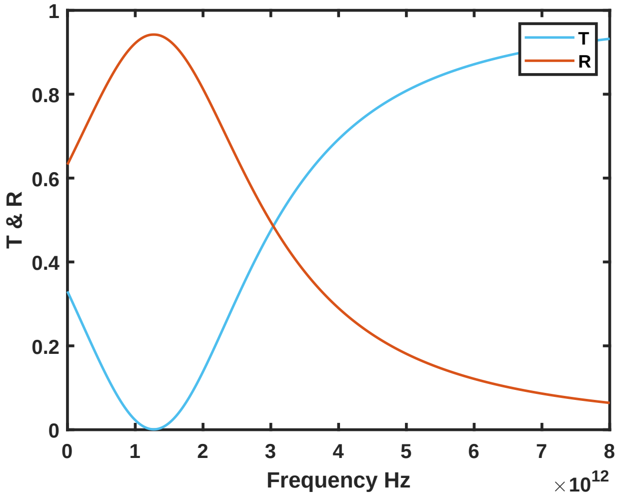

(b) B = 4 Tesla.

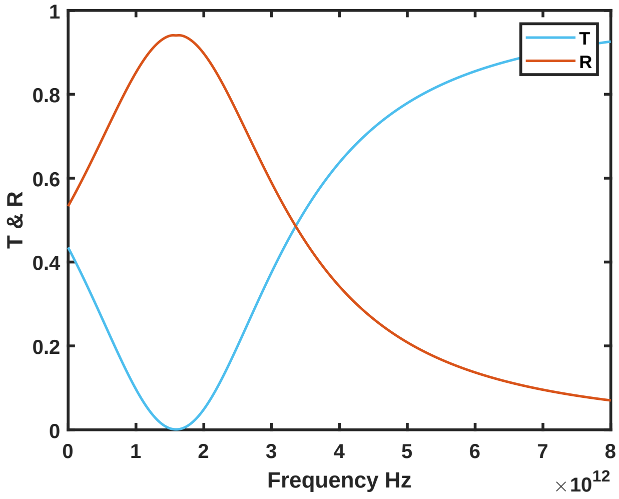

(c) B = 5 Tesla.

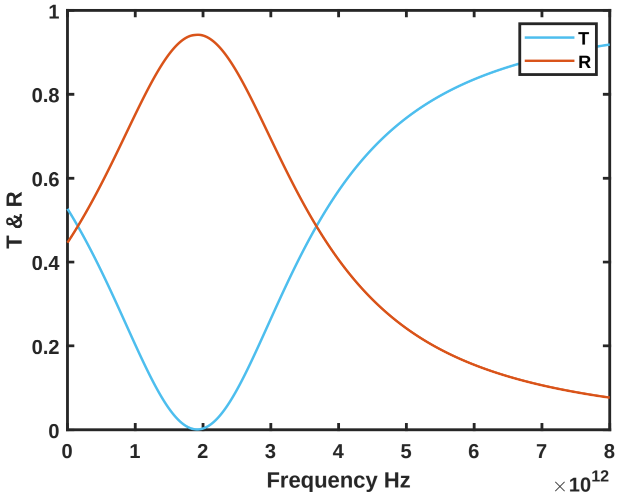

(d) B = 6 Tesla.

Supplementary figure S5: Transmission and reflection for left handed polarization, chemical potential: $0.5ev$, $T =$ 10K

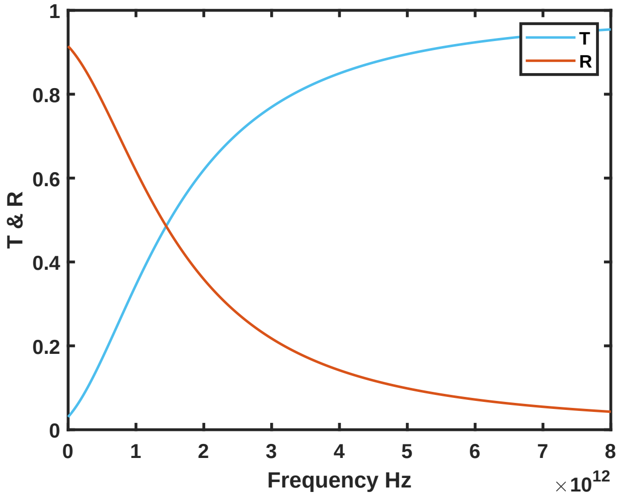

(a) B = 1 Tesla.

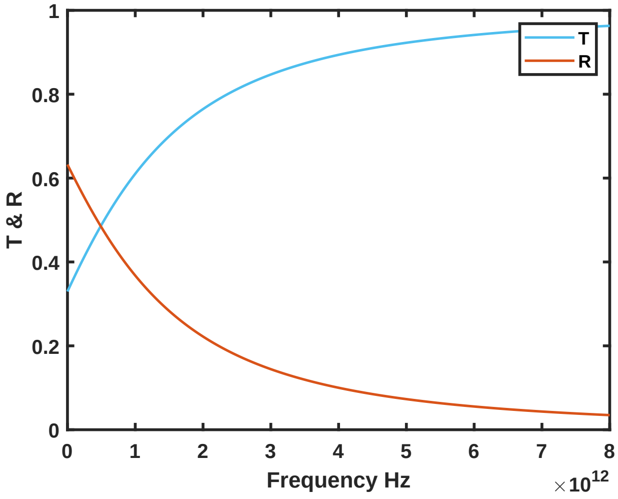

(b) B = 4 Tesla.

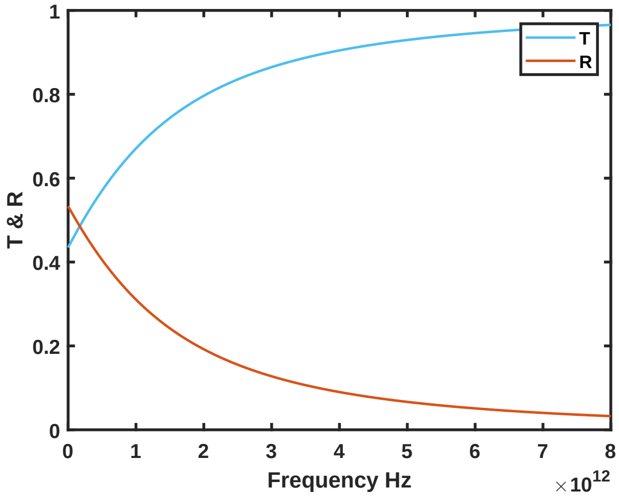

(c) B = 5 Tesla.

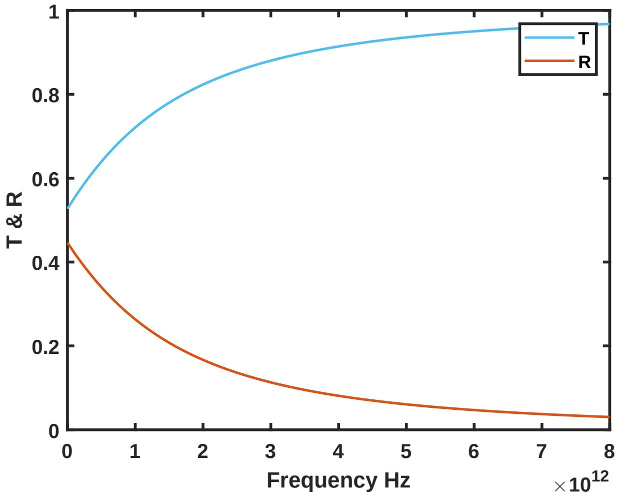

(d) B = 6 Tesla.

Supplementary figure S6: Transmission and reflection for right handed polarization, chemical potential: $0.5ev$, $T =$ 10K

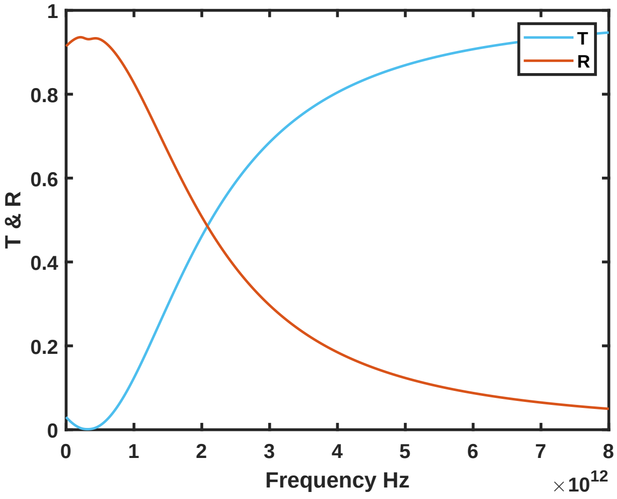

(a) B = 1 Tesla.

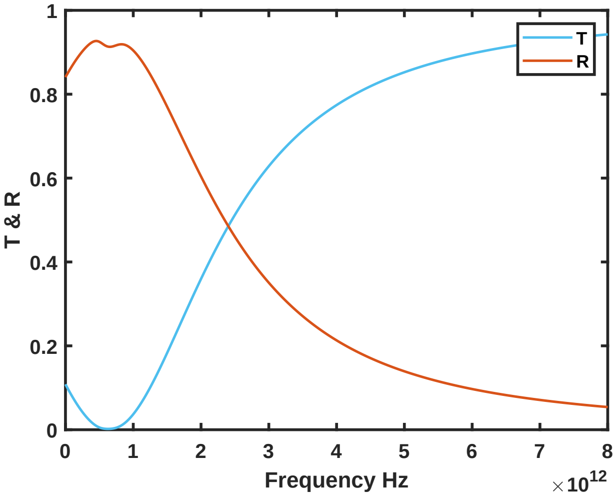

(b) B = 4 Tesla.

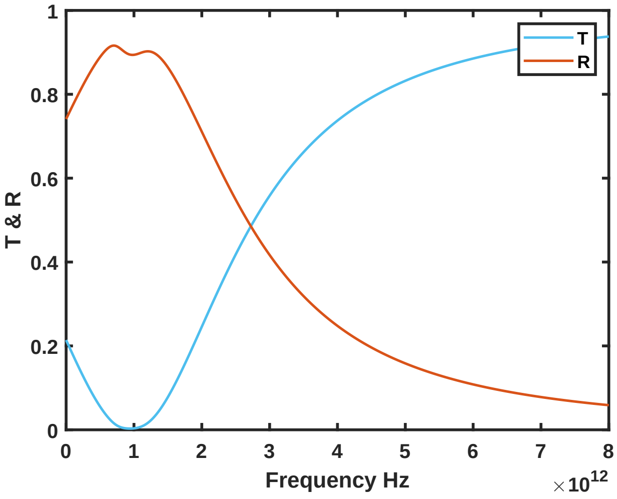

(c) B = 5 Tesla.

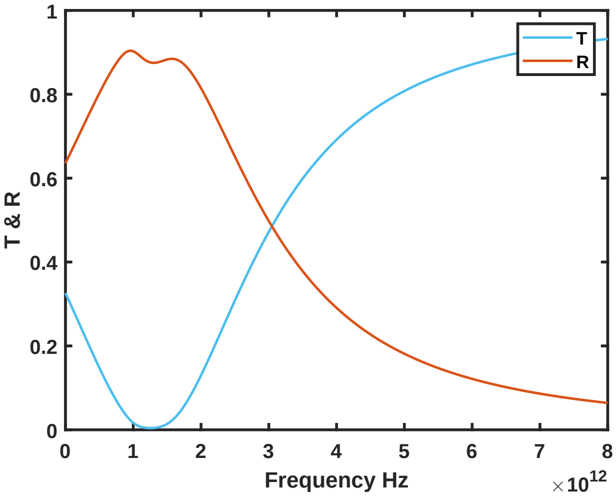

(d) B = 6 Tesla.

Supplementary figure S7: Transmission and reflection for left handed polarization, chemical potential: $0.5ev$, $T =$ 300K

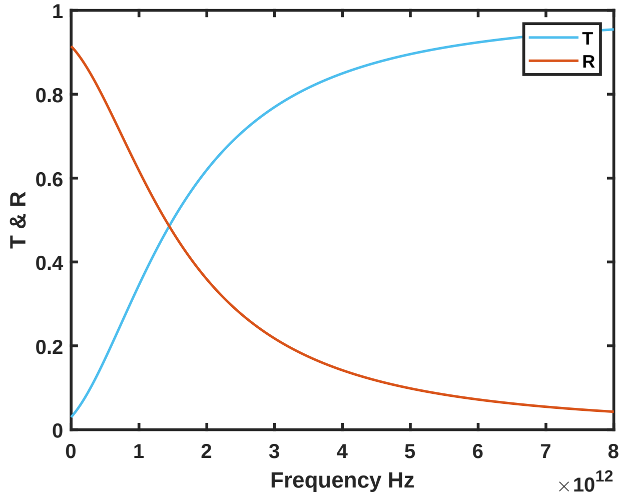

(a) B = 1 Tesla.

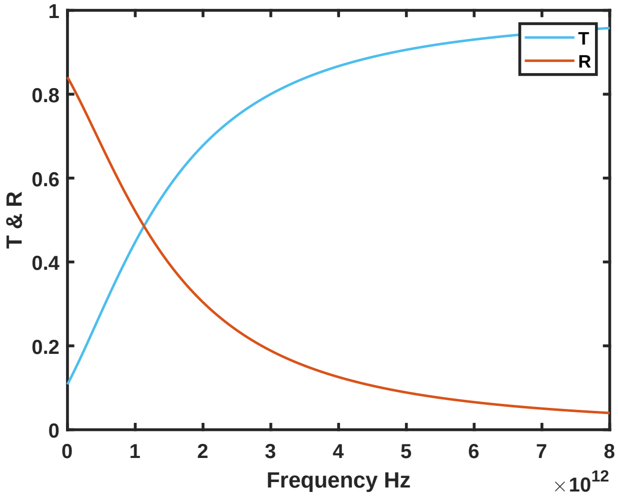

(b) B = 4 Tesla.

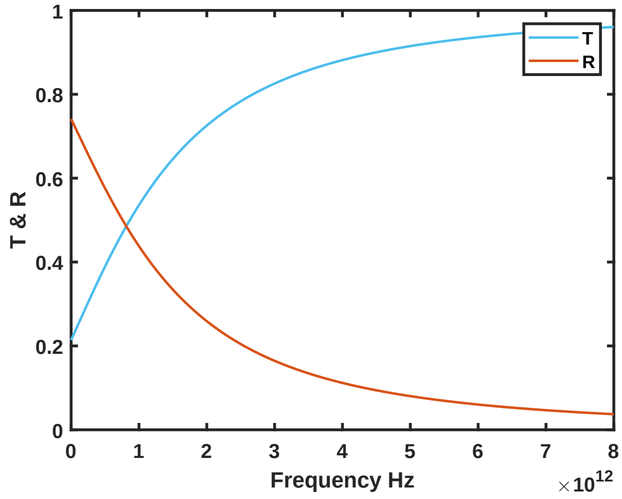

(c) B = 5 Tesla.

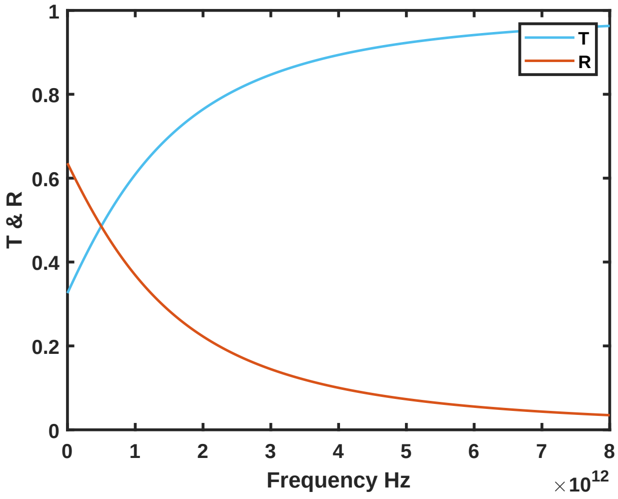

(d) B = 6 Tesla.

Supplementary figure S8: Transmission and reflection for right handed polarization, chemical potential: $0.5ev$, $T =$ 300K

II. CPA FREQUENCY AGAINST APPLIED MAGNETIC FIELD AND VARIED CHEMICAL POTENTIAL

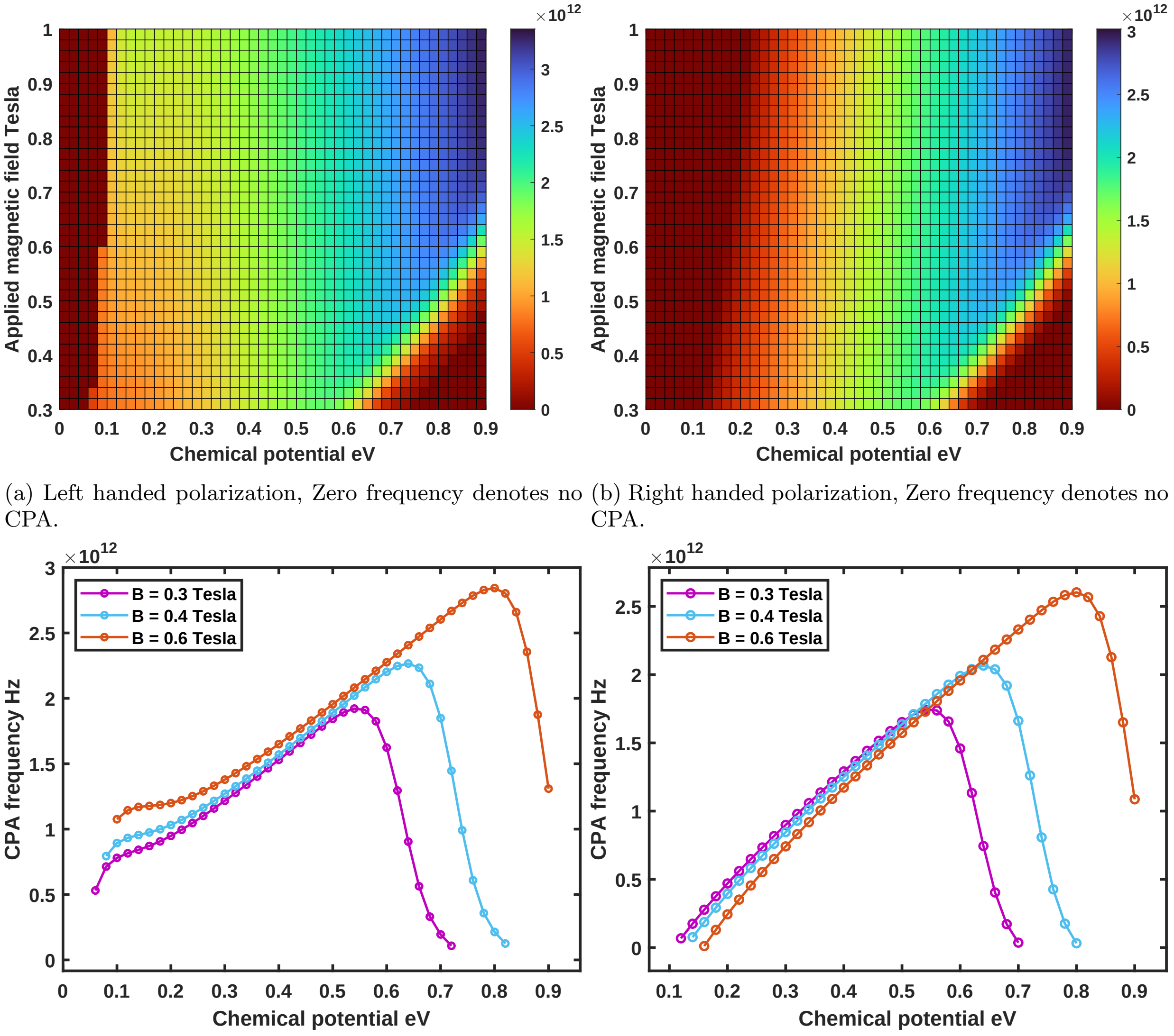

(a) Left handed polarization, Zero frequency denotes no CPA.

(b) Right handed polarization, Zero frequency denotes no CPA.

(c) Left handed polarization.

(d) Right handed polarization.

Supplementary figure S9: CPA frequency against magnetic field and different chemical potential

III. COHERENT ABSORPTION

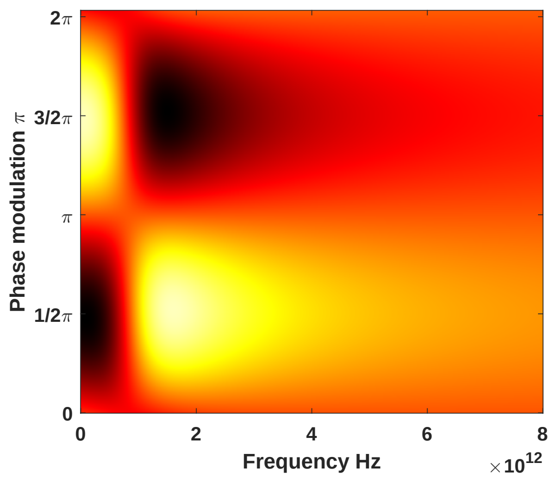

(a) B = 1 Tesla.

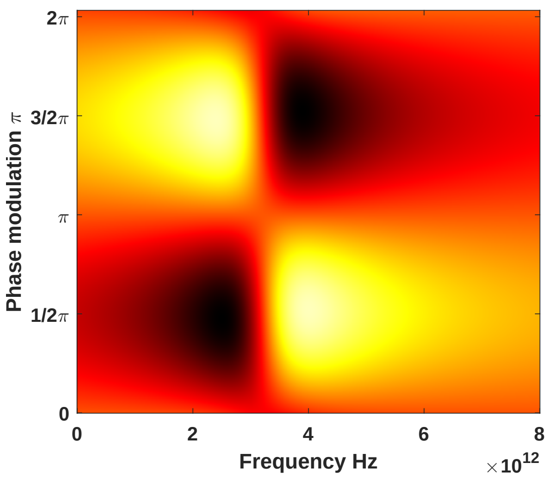

(b) B = 4 Tesla.

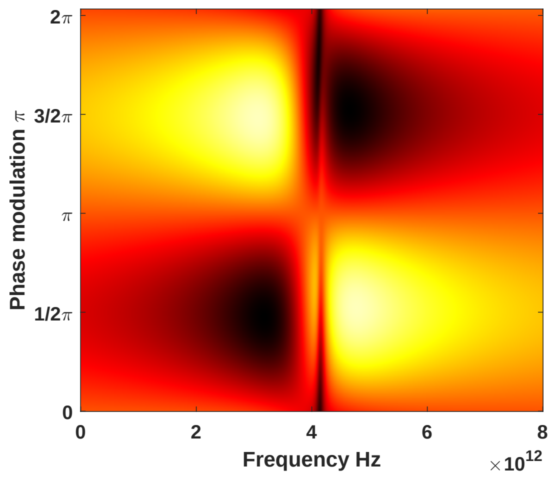

(c) B = 5 Tesla.

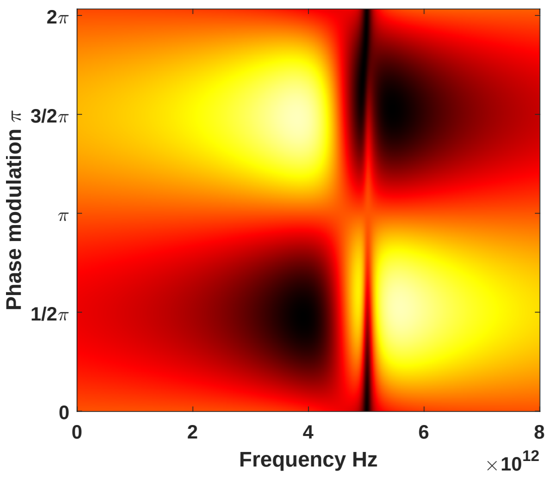

(d) B = 6 Tesla.

Supplementary figure S10: Coherent absorption for left handed polarization, 1 denotes the maximum absorption coefficient, chemical potential: $0.2ev$, $T = 10$K

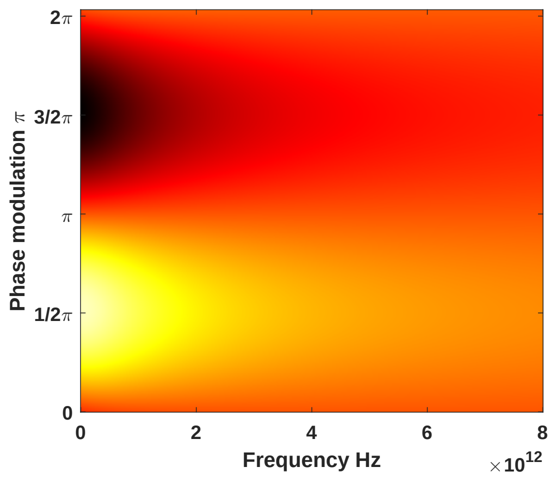

(a) B = 1 Tesla.

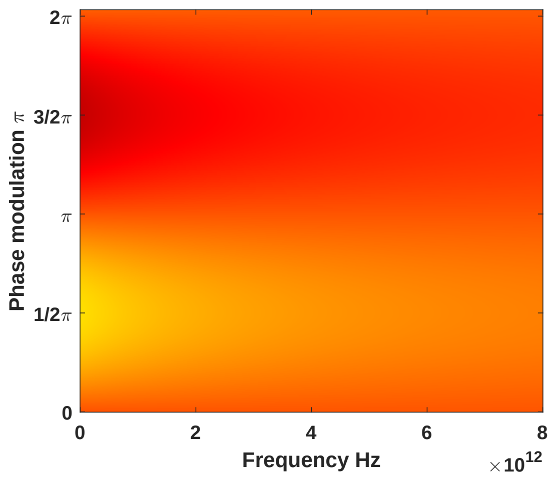

(b) B = 4 Tesla.

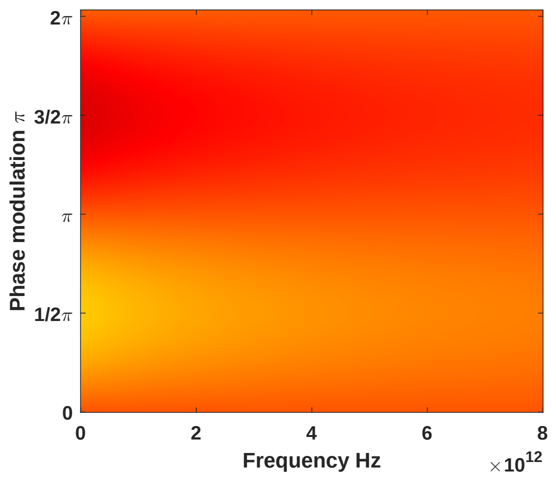

(c) B = 5 Tesla.

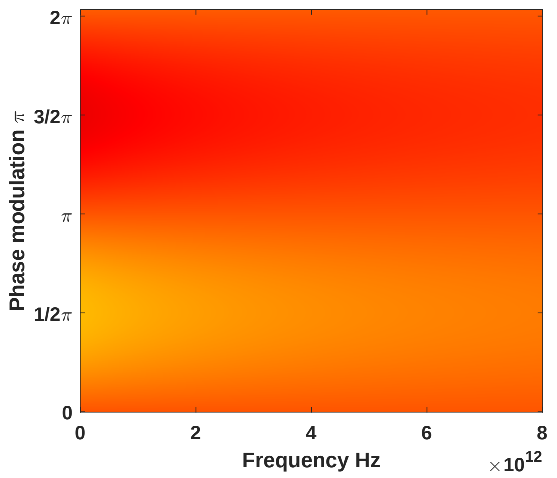

(d) B = 6 Tesla.

Supplementary figure S11: Coherent absorption for right handed polarization, 1 denotes the maximum absorption coefficient, chemical potential: $0.2ev$, T = 10K

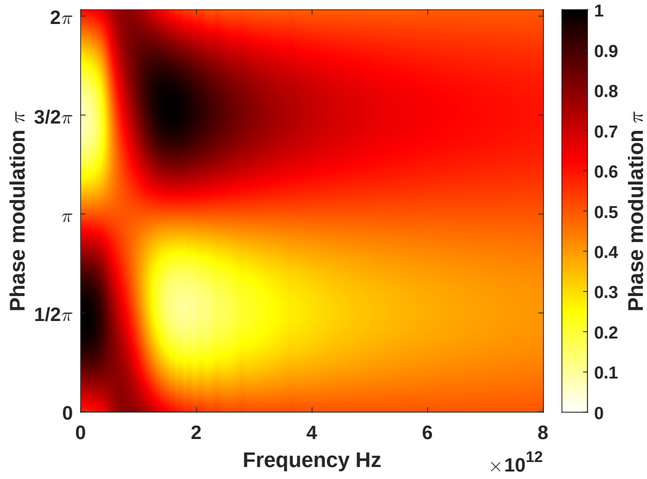

(a) B = 1 Tesla.

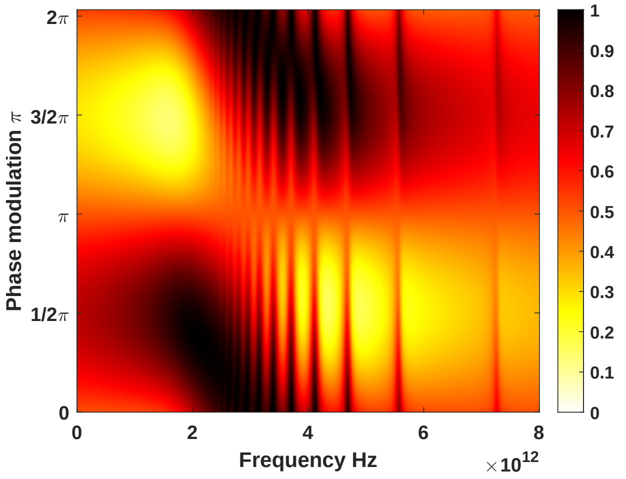

(b) B = 4 Tesla.

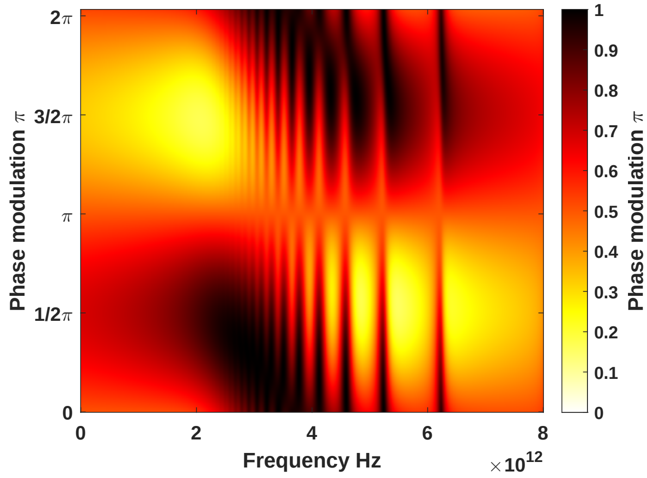

(c) B = 5 Tesla.

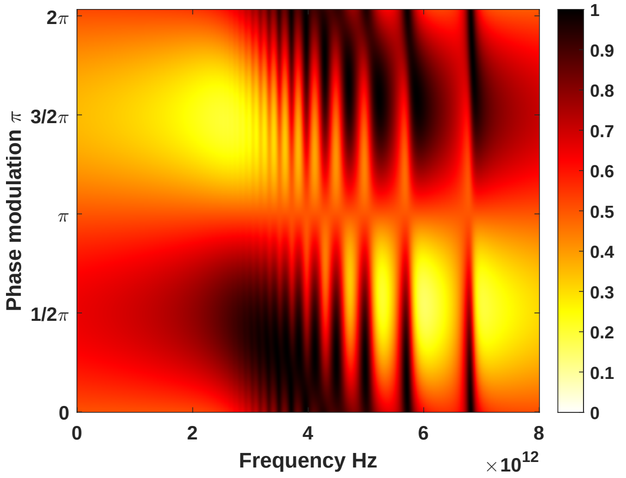

(d) B = 6 Tesla.

Supplementary figure S12: Coherent absorption for left handed polarization, 1 denotes the maximum absorption coefficient, chemical potential: $0.2ev$, $T = 300$K

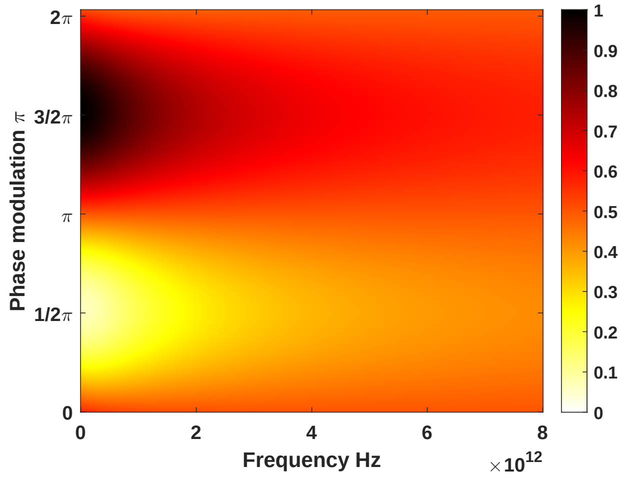

(a) B = 1 Tesla.

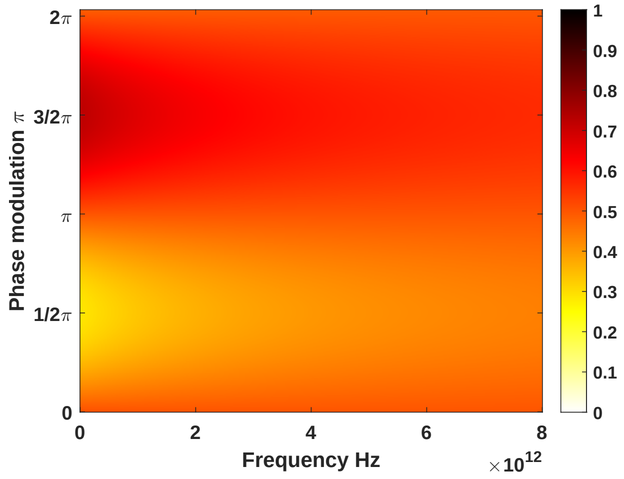

(b) B = 4 Tesla.

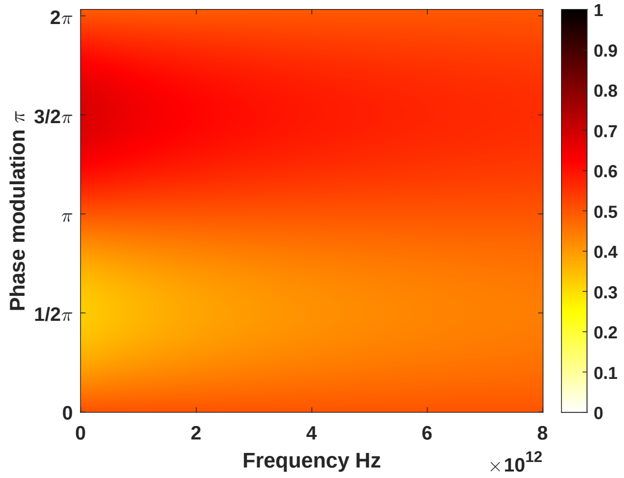

(c) B = 5 Tesla.

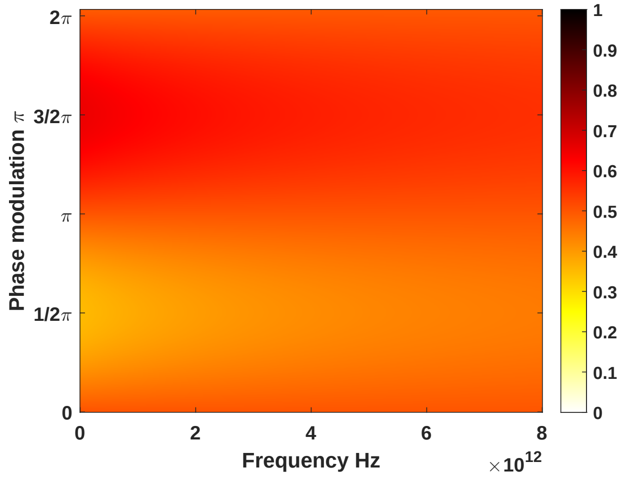

(d) B = 6 Tesla.

Supplementary figure S13: Coherent absorption for right handed polarization, 1 denotes the maximum absorption coefficient, chemical potential: $0.2ev$, $T = 300$K

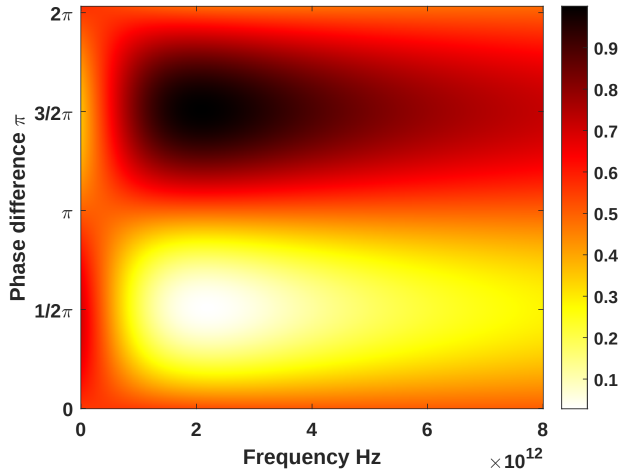

(a) B = 1 Tesla.

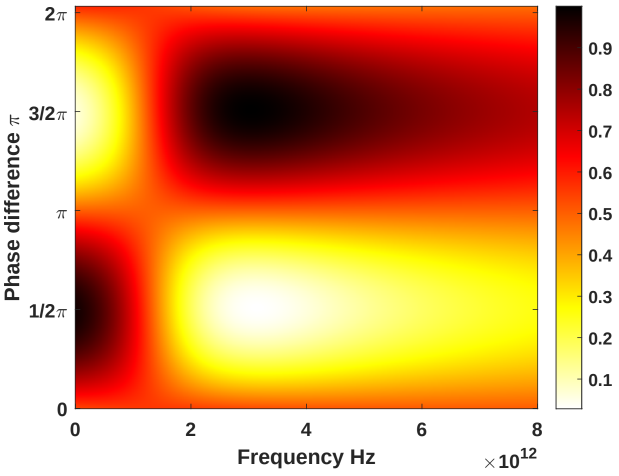

(b) B = 4 Tesla.

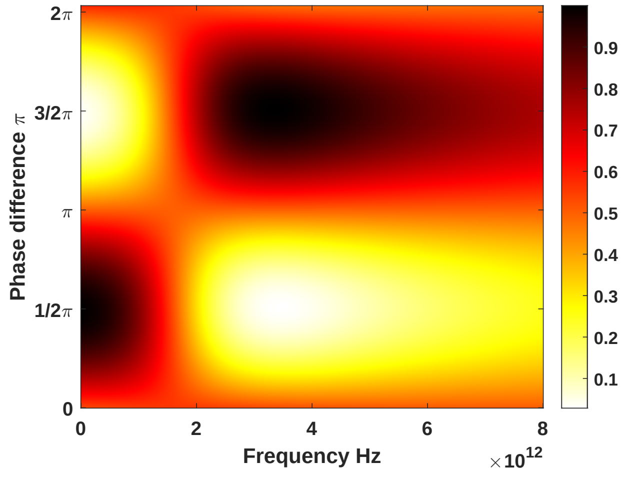

(c) B = 5 Tesla.

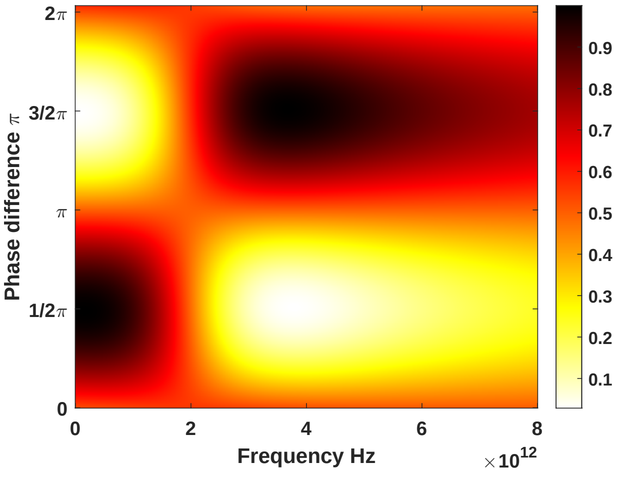

(d) B = 6 Tesla.

Supplementary figure S14: Coherent absorption for left handed polarization, 1 denotes the maximum absorption coefficient, chemical potential: $0.5ev$, $T = 10$K

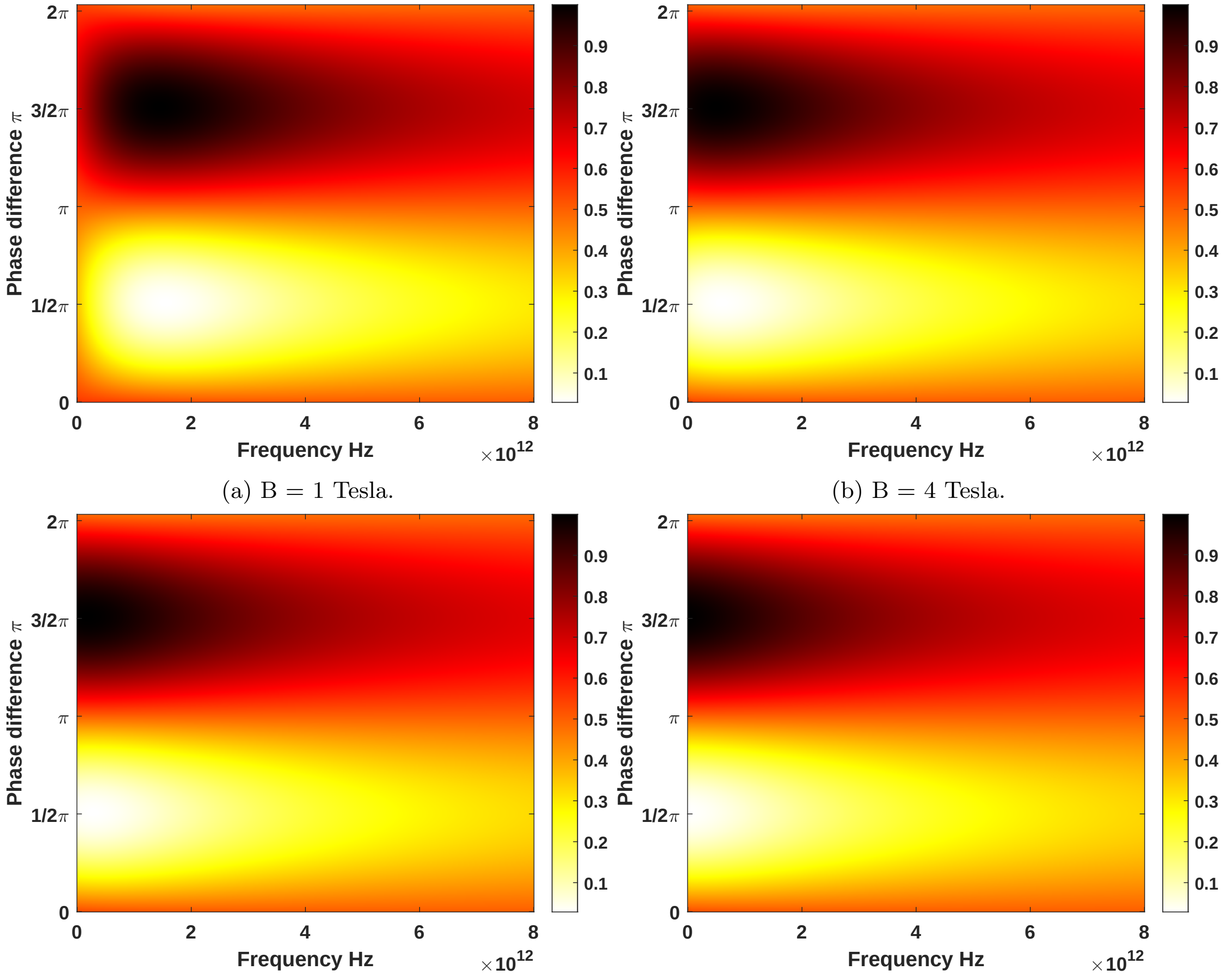

(a) B = 1 Tesla.

(b) B = 4 Tesla.

(c) B = 5 Tesla.

(d) B = 6 Tesla.

Supplementary figure S15: Coherent absorption for right handed polarization, 1 denotes the maximum absorption coefficient, chemical potential: $0.5ev$, $T = 10$K

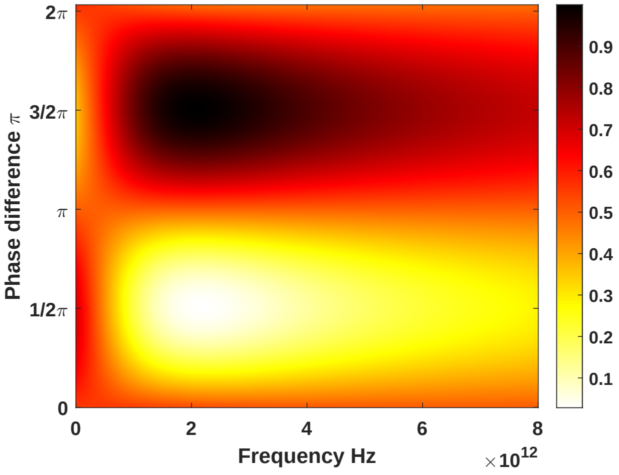

(a) B = 1 Tesla.

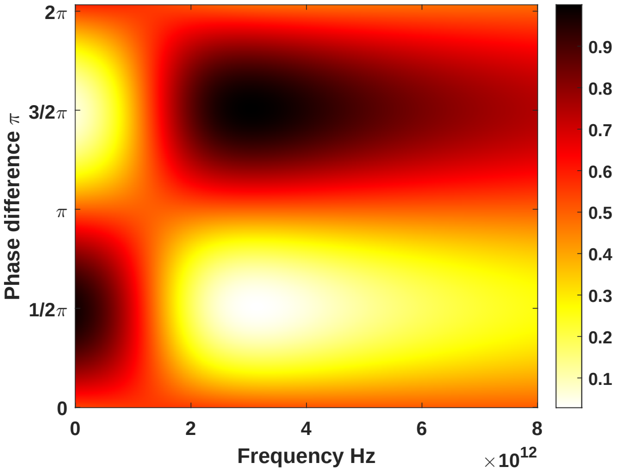

(b) B = 4 Tesla.

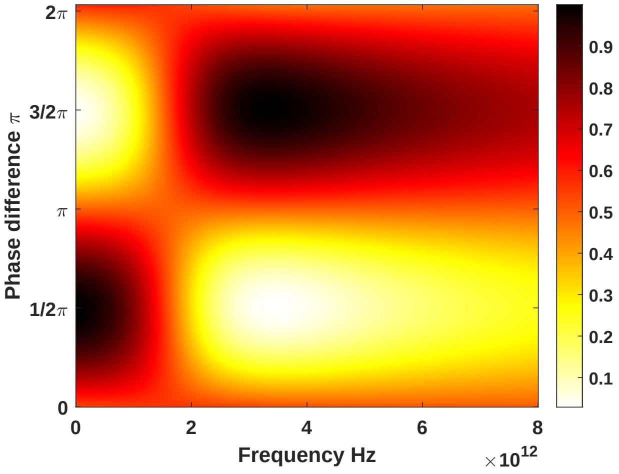

(c) B = 5 Tesla.

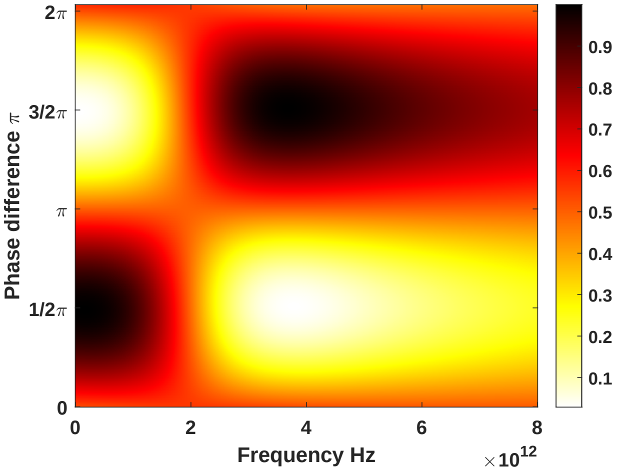

(d) B = 6 Tesla.

Supplementary figure S16: Coherent absorption for left handed polarization, 1 denotes the maximum absorption coefficient, chemical potential: $0.5ev$, $T = 300$K

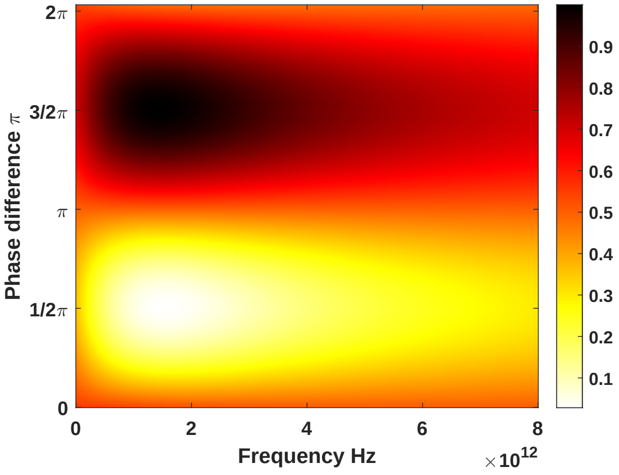

(a) B = 1 Tesla.

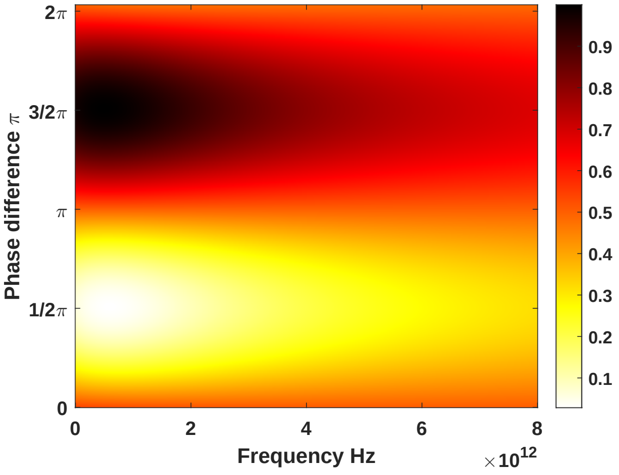

(b) B = 4 Tesla.

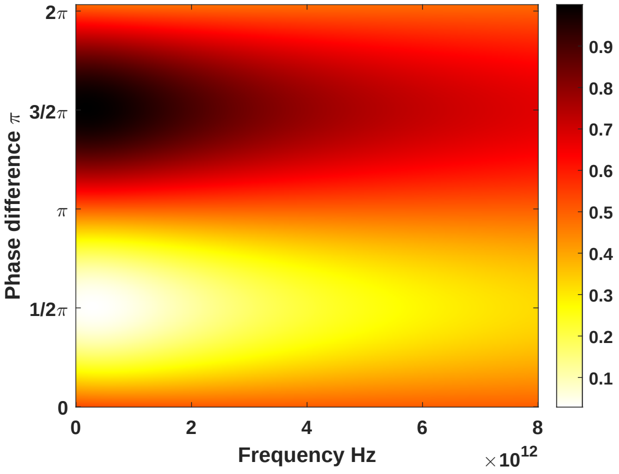

(c) B = 5 Tesla.

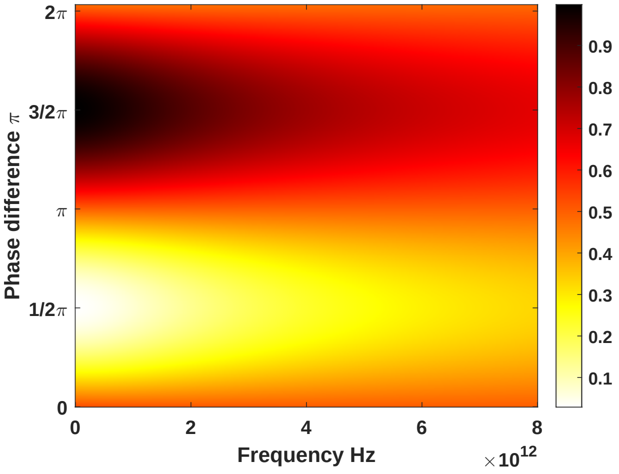

(d) B = 6 Tesla.

Supplementary figure S17: Coherent absorption for right handed polarization, 1 denotes the maximum absorption coefficient, chemical potential: $0.5ev$, $T = 300$K
